# Random Zero-Sum Dynamic Games on Infinite Directed Graphs

**DOI:** 10.1007/s13235-025-00636-4

**Published:** 2025-03-21

**Authors:** Luc Attia, Lyuben Lichev, Dieter Mitsche, Raimundo Saona, Bruno Ziliotto

**Affiliations:** 1 CEREMADE, Paris Dauphine University, Paris, France; 2 University Jean Monnet, Saint-Etienne, France; 3 Institute of Mathematics and Informatics, Bulgarian Academy of Sciences, Sofia, Bulgaria; 4 Institute for Mathematical and Computational Engineering, Pontifícia Universidad Católica, Santiago, Chile; 5 Institute of Science and Technology Austria, Klosterneuburg, Austria; 6 CNRS UMR 5219, Toulouse, France; 7 Toulouse School of Economics, Toulouse, France; 8 Université Toulouse Capitole, Toulouse, France; 9 Institut de Mathématiques de Toulouse, Toulouse, France

**Keywords:** Zero-sum games, Dynamic games, Random games, Directed graphs, 91A05, 91A10, 91A25, 91A50, 05C05, 05C63

## Abstract

We consider random two-player zero-sum dynamic games with perfect information on a class of infinite directed graphs. Starting from a fixed vertex, the players take turns to move a token along the edges of the graph. Every vertex is assigned a payoff known in advance by both players. Every time the token visits a vertex, Player 2 pays Player 1 the corresponding payoff. We consider a distribution over such games by assigning i.i.d. payoffs to the vertices. On the one hand, for acyclic directed graphs of bounded degree and sub-exponential expansion, we show that, when the duration of the game tends to infinity, the value converges almost surely to a constant at an exponential rate dominated in terms of the expansion. On the other hand, for the infinite *d*-ary tree (that does not fall into the previous class of graphs), we show convergence at a double-exponential rate.

## Introduction

### Dynamic Games on Graphs

Zero-sum dynamic games with perfect information played on graphs [1] provide a powerful mathematical framework to analyze several important problems in mathematics and computer science. They correspond to stochastic games [2] with the restrictions that the transitions are deterministic and players play in turns. Considering a graph where every vertex has an outgoing edge, the game starts with a token at a vertex. Then, two players, who both know the position of the token at all times, alternate in choosing to move it along an outgoing edge to a neighboring vertex. Each vertex is assigned a uniformly bounded number called *payoff* and every time the token visits a vertex, Player 2 pays Player 1 its payoff. As the game evolves, the token moves from one vertex to another indefinitely, thus generating a sequence of payoffs.

### Limit Value

In the *n*-stage game, the objective of Player 1 is to maximize the mean payoff over *n* stages. Similarly, Player 2 aims to minimize this mean. The *value* of the *n*-stage game, denoted by $v_n$, is the maximum mean payoff Player 1 can guarantee irrespective of the actions of Player 2. Studying the behavior of dynamic games, or in general stochastic games, when the duration of the game tends to infinity, has been the subject of intense research over the last fifty years. One question that has received particular interest is the existence of a limit of the sequence $(v_n)_{n \ge 1}$ as *n* tends to infinity. When it exists, this limit can be interpreted as the asymptotic mean payoff of Player 1 over an infinitely long game, and it thus stands out as a fundamental concept in the theory of stochastic games with long duration. A seminal result of Bewley and Kohlberg [3] states that, when the state space and the action sets are finite, the sequence $(v_n)_{n \ge 1}$ converges even for general stochastic games. This result has been extended in many directions, ranging from models with partial observation of the state and actions through models with unknown duration to models with objective functions other than mean payoff, see [4–7]. Proving convergence of $(v_n)_{n \ge 1}$ turns out to be a particularly delicate task when the state space is infinite. Indeed, positive results are scarce (see [8, 9] for some recent advances) and counterexamples have been found [10]. When the action space is infinite, there are positive and negative results [11].

### Random Dynamic Games

Recently, a class of random zero-sum dynamic games on infinite graphs with vertices in $\mathbb {Z}^d$ has been introduced under the name of *percolation games* [12]. In this model, as in usual dynamic games, each vertex is assigned a uniformly bounded payoff, and these values are known by the players before the game starts. The authors study a distribution over such games given by assigning i.i.d. random payoffs to the vertices. Then, the asymptotic behavior of the random value of the *n*-stage game, denoted by $V_n$, is studied. It is shown in [12] that, under the assumption that payoffs are uniformly bounded and every action increases the projection of the position of the token onto some axis, $(V_n)_{n \ge 1}$ converges almost surely to a deterministic limit value. Moreover, they provide exponential concentration estimates for $(V_n)_{n \ge 1}$ around the limit of their expectation. One important takeaway message of this result is that equipping the state space with a particular graph structure (e.g., $\mathbb {Z}^d$) and assuming that payoffs have some probabilistic regularity (e.g., i.i.d. random variables) can ensure the existence of the limit of $(V_n)_{n \ge 1}$ even for an infinite graph.

### Contributions

In this paper, we extend the results in [12] to a class of graphs that is fairly more general than $\mathbb {Z}^d$. In more detail, we introduce *directed games*, a class of games on acyclic directed graphs $\Gamma$ where players move the token along the edges of $\Gamma$. On the one hand, under certain assumptions of weak transitivity and sub-exponential growth of $\Gamma$, we prove that $(V_n)_{n \ge 1}$ is exponentially concentrated around a given deterministic limit value (so, in particular, $(V_n)_{n \ge 1}$ a.s. converges to that value) and relate the convergence rate to the expansion of the graph (see Theorem 10). On the other hand, we consider the infinite *d*-ary tree where each vertex has exactly $d \ge 2$ children and every edge is directed from the parent to the child. These graphs do not belong to the previous class of games due to their exponential expansion away from the root. In this case, we show a stronger double-exponential concentration of the random variables $(V_n)_{n \ge 1}$ around their respective expectations.

In contrast to [12], the graphs that we consider here do not have to be transitive and, more importantly, they do not necessarily grow polynomially. This leads to several differences in the proof techniques. On a technical level, the novelty of our paper lies in treating a significantly more general class of graphs than the class of percolation games [12] while providing sharper concentration estimates. Note that the game being weakly transitive, and not simply transitive, introduces additional technicalities. Lastly, the proof of the second result on *d*-ary trees significantly differs from the arguments used in [12]: it is based on a pruning argument to avoid certain subtrees.

### Related Work

In our model, the payoffs associated with the states are the only random variables, and their realizations are known to both players before the game starts. Once the payoffs are revealed, the players play a deterministic game. Distributions over games, such as in our model of random dynamic games, have recently received growing attention. For example, studying random zero-sum games on graphs and their long-term behavior goes beyond the game theory community [13–16]. Indeed, on the one hand, a class of random games has been used to solve a well-known open problem regarding Probabilistic Finite Automata [17] (see [18] for an extension). On the other hand, the class of games studied in [12] has served as a toy model for contributing to a well-studied problem in the theory of PDEs called *stochastic homogenization* (see [12, Section 4] and [19, 20]).

### Outline

This paper is organized as follows. In Sect. 2, we formally define our model and state our main results. In Sect. 3, we state some preliminary results for later use. Then, Sects. 4 and 5 are dedicated to the proofs of our main results on controlled expansion graphs and *d*-ary trees, respectively. Finally, in Sect. 6, we show that our results cover all previously defined percolation games.

## Model and Main Results

### The Model

A *directed game* is a dynamic game that consists of a locally finite directed graph $\Gamma$ with infinite countable vertex set *Z* called the *state space*, an initial state $z_0 \in Z$ and a collection of independent and identically distributed (i.i.d.) random variables $(G_z)_{z \in Z}$ called *payoffs* defined over a probability space $(\Omega , {\mathcal {F}}, \mathbb {P})$. We assume that $\Gamma$ has uniformly bounded degrees and contains neither directed cycles nor vertices with out-degree zero. The game is played by two players called Player 1 and Player 2. At the start of the game, the random payoffs $(G_{z})_{z \in Z}$ are sampled and $(G_{z}(\omega ))_{z \in Z}$ are presented to both players, who thus obtain perfect information. Then, a token is placed at the initial state $z_0$. For every integer $i \ge 0$, stage *i* proceeds as follows. Given that the token is positioned at a state $z \in Z$,

- if *i* is even, Player 1 moves the token to an out-neighbor $z'$ of *z* in $\Gamma$, and
- if *i* is odd, Player 2 moves the token to an out-neighbor $z'$ of *z* in $\Gamma$.

After the token has been moved from *z* to $z'$, Player 1 receives the payoff $G_{z'}(\omega )$ from Player 2 and stage *i* ends. We are mostly interested in the *n*-*stage game* consisting of the first *n* stages for (typically large) integers *n*.

While the setting in  [12] also considers players making moves alternately and with perfect information, one stage there consists of a move of the first and a move of the second player. This is not a fundamental difference and separating every move in a different stage is only done to enhance the clarity of the exposition.

A *strategy* of Player 1 (resp. Player 2) is a function $\sigma :\Omega \times \bigcup _{m \ge 0} Z^{2 m + 1} \rightarrow Z$ (respectively $\tau :\Omega \times \bigcup _{m \ge 0} Z^{2m+2} \rightarrow Z$) with the property that, for every $m \ge 0$ and $(z_0,z_1,\dots ,z_{2 m + 1}) \in Z^{2 m + 2}$, $\Gamma$ contains the edge from $z_{2 m}$ to $\sigma (\omega , z_0, \dots , z_{2 m})$ (resp. from $z_{2 m + 1}$ to $\tau (\omega , z_0, \dots , z_{2 m + 1})$). We denote by $\Sigma$ the collection of all strategies for Player 1 and by ${\mathcal {T}}$ the collection of all strategies for Player 2.

Given a pair of strategies $(\sigma , \tau )\in \Sigma \times {\mathcal {T}}$, we define inductively the *trajectory of the token* by setting $z_{2 i + 1}:= \sigma (\omega , z_0, \dots , z_{2 i})$ and $z_{2 i + 2}:= \tau (\omega , z_0, \dots , z_{2 i + 1})$ for every $i \ge 0$. This allows us to define the *n*-*stage payoff function* $\gamma ^{z_0}_n :\Omega \times \Sigma \times {\mathcal {T}} \rightarrow \mathbb {R}$ by setting

$$\begin{aligned} \gamma ^{z_0}_n(\omega , \sigma , \tau ):= \frac{1}{n} \sum _{i = 1}^{n} G_{z_i}(\omega ) . \end{aligned}$$

Recall that the directed graph $\Gamma$ is locally finite. Therefore, the *n*-stage game with initial state $z_0 = z \in Z$ is a perfect information finite game whose value $V_n :\Omega \times Z \rightarrow \mathbb {R}$ is defined as usual by

$$\begin{aligned} V_n(\omega , z):= \max _{\sigma \in \Sigma } \min _{\tau \in {\mathcal {T}}} \gamma ^{z}_n(\omega , \sigma , \tau ) = \min _{\tau \in {\mathcal {T}}} \max _{\sigma \in \Sigma } \gamma ^{z}_n(\omega , \sigma , \tau ) , \end{aligned}$$

where the equality between maxmin and minmax is given by Kuhn’s theorem [21, Theorem 1].

Moreover, we will say that a strategy $\sigma \in \Sigma$ (resp. $\tau \in \mathcal T$) is *optimal* for the *n*-stage game (starting from *z*) if, for all realization of the payoffs $\omega \in \Omega$, the strategy $\sigma$ maximizes $\min _{\tau \in {\mathcal {T}}} \gamma _n^z(\omega , \,\cdot \,, \tau )$ over $\Sigma$ (resp. if $\tau$ minimizes $\max _{\sigma \in \Sigma } \gamma _n^z(\omega , \sigma , \,\cdot \, )$ over ${\mathcal {T}}$).

A classic question in the game-theoretic literature initiated by [3] is to ask for the convergence of the *n*-stage value as *n* grows to infinity. Since payoffs are random, $V_n$ is a random variable. Therefore, we are interested in whether the sequence $(V_n)_{n \ge 1}$ converges a.s. to a constant. If no further assumptions are imposed, $(V_n)_{n \ge 1}$ does not necessarily converge, as the following example shows.

#### Example 1

For all integers $m\ge 0$, set $n_m:= 2^{2^{2m}}$ and $n'_m:= 2^{2^{2m+1}}$ and consider the case where $\Gamma$ is a directed tree (all edges being directed away from the root) where each node of even height has only one child, while each node with odd height *k* has two children if $k=1$ or $k\in [n_m,n_m')$ for some $m\ge 0$, and it has only one child if $k\in [n_m',n_{m+1})$. Moreover, let the payoffs be i.i.d. Bernoulli random variables with parameter 1/2. In particular, for every $m\ge 1$, in the $n_m$-stage game, Player 2 has only one choice most of the time, while in the $n_m'$-stage game, she has two choices most of the time. Since Player 2 can pick a vertex with payoff 0 (if it is present), and pick an available vertex otherwise, one can show that a.s.

$$\begin{aligned} \limsup _{m\rightarrow \infty } V_{n_m'}\le \frac{3}{8} < \frac{1}{2} = \lim _{m\rightarrow \infty } V_{n_m} . \end{aligned}$$

Indeed, while Player 1 never has a choice in the $n_m'$-stage game (implying that the mean payoff over the odd states visited by the token a.s. converges to 1/2), Player 2 can ensure with the above strategy that the mean payoff over the even states visited by the token a.s. converges to 1/4, which yields that a.s. $\limsup _{m\rightarrow \infty } V_{n_m'}\le 3/8$. At the same time, for every $\varepsilon > 0$, Chernoff’s bound for the Binomial distribution $\textrm{Bin}(n_m, 1/2)$ and a union bound over the $O(2^{n_{m-1}'})$ vertices at level $n_m$ in $\Gamma$ shows that $V_{n_m}$ is in the interval $[1/2-\varepsilon , 1/2+\varepsilon ]$ with probability very close to 1. In particular, a.s. $(V_n)_{n \ge 1}$ does not converge. Therefore, to ensure convergence, we will need further structural assumptions on the graph.

Before turning to our results, we provide some vocabulary. Given a vertex $z\in Z$, a *descendant* of *z* (in $\Gamma$) is a vertex that can be reached from *z* by a directed path in $\Gamma$. We say that *z* and $z'$ are *equivalent* if the two subgraphs of $\Gamma$ induced by the descendants of *z* and by the descendants of $z'$, respectively, are isomorphic (as directed graphs).

#### Definition 2

The graph $\Gamma$ is *weakly transitive* if there is a state $z^*$ and an integer $M \ge 0$ such that the following holds: for each state $z\in Z$, in the game with initial state $z_0 = z$, each player has a strategy that, independently of the moves of the opponent, ensures that the token is placed at a state equivalent to $z^*$ after an even number of $\ell \le M$ stages.

Note that all vertex-transitive graphs are weakly transitive with $M = 0$. We also remark that the parity of $\ell$ is important to ensure that, when the token reaches $z^*$, it is Player 1’s turn to make a move. For similar notions of generalized vertex-transitive graphs, see for example [22].

In the remainder of the paper, we always assume that $\Gamma$ is weakly transitive and drop the dependence of the random variables on $\omega$. The next two subsections present two types of directed games used in our main results.

#### Weakly Transitive Games with Sub-Exponential Expansion

One of the main difficulties in the analysis of the asymptotic behavior of $(V_n)_{n \ge 1}$ is to make use of the independence of the payoffs $(G_z)_z$. To this end, we use the partial order introduced by the directed graph $\Gamma$ on the state space. More formally, let $z_1, z_2 \in Z$. We say that $z_1 \preceq z_2$ if $z_2$ is a descendant of $z_1$. Then, for all $z \in Z$ and $n \ge 1$, denote $Z_n(z)$ the set of *reachable states* from *z* in exactly *n* steps, and $Z^{(n)}(z)$ the ones reachable in at most *n* steps. Since $\Gamma$ is locally finite, $Z^{(n)}(z)$ is a finite partially ordered set. Denote *h*(*n*, *z*) its height, that is, the longest path in $Z^{(n)}(z)$. Note that, starting from *z*, after *h*(*n*, *z*) stages, the game never returns to a state in $Z^{(n)}(z)$ as $\Gamma$ is acyclic, thus separating $Z^{(n)}(z)$ from the future of the game. Hence, *h*(*n*, *z*) can be considered as the waiting time after which the future of the game becomes independent of $Z^{(n)}(z)$. In this regard, we define the largest possible waiting time, which we call the *transient speed function*, as

$$\begin{aligned} h :n \in \mathbb {N}\mapsto \sup _{z \in Z} h(n, z) \in \mathbb {N}\cup \{\infty \} . \end{aligned}$$

We note that Mirsky’s theorem (see [23, Theorem 2]) gives us a dual interpretation of *h*(*n*, *z*) as the minimum number of antichains needed to partition $Z^{(n)}(z)$. Thus, *h*(*n*) may be seen as an upper bound on these minima. Following this point of view, we expect to obtain a concentration inequality parameterized by *h*(*n*) since the payoffs are i.i.d. In our analysis, this concentration has to be sufficiently strong to overcome a union bound over all possible states from which the game may continue, which is quantified by the growth of $Z^{(n)}(z)$. Formally, we will handle concentration inequalities bounding from above $\mathbb {P}( |X - \mathbb {E}[X] | \ge t )$ for suitable random variables *X*, so we define the following key function $\psi :\mathbb {N}\times (0, \infty ) \rightarrow \mathbb {R}$ by

$$\begin{aligned} \psi (n, t):= \exp \left( -\frac{t^2 n^2}{2h(n)} \right) \max _{z \in Z} |Z^{(2n)}(z)| . \end{aligned}$$

Lemma 14 later essentially proves that $\mathbb {P}( |V_n(z_0) - \mathbb {E}[V_n(z_0)]| \ge t) \le 2 \exp \left( -\frac{t^2n^2}{2 h(n)}\right)$. When proving the convergence of the value, we need to consider every reachable state after at most 2*n* steps, which leads to an expression of the form of $\psi (n, \varepsilon _n)$.

Our main goal is to analyze directed games in which the size of the sets $Z^{(n)}(z)$ does not grow too fast as *n* grows to infinity, which we formalize as follows.

##### Definition 3

($\delta$-transient games) For a fixed $\delta > 0$, a directed game on a graph $\Gamma$ with vertex set *Z* is called $\delta$-*transient* if there exists a sequence $(\varepsilon _n)_{n \ge 1}$ of positive real numbers such that

$$\begin{aligned} \varepsilon _n + \psi (n, \varepsilon _n) = O(n^{-\delta }) , \end{aligned}$$

where the asymptotic notation is with respect to $n\rightarrow \infty$. In other words, there is a sequence $(\varepsilon _n)_{n \ge 1}$ that decreases to zero at least as fast as $n^{-\delta }$ such that the expression $\psi (n, \varepsilon _n)$ also decreases to zero at least that fast. Recalling that $\psi (n, \varepsilon _n)$ will serve as an upper bound for expressions of the form $\mathbb {P}( |X - \mathbb {E}[X] | \ge \varepsilon _n)$, this speed of convergence quantifies the fact that the concentration is strong enough to make the gap $\varepsilon _n$ small as long as *n* is big enough.

##### Remark 4

The concept of $\delta$-transient games is only relevant for $\delta \in (0, 1/2)$ because there is no directed game that is $\delta$-transient for some $\delta \ge 1/2$. Indeed, consider a $\delta$-transient game. By Definition 3, we have that $(\psi (n, \varepsilon _n))_{n \ge 1}$ converges to zero. In particular, we have that $\varepsilon _n^2 n^2 / h(n) \rightarrow \infty$. Since $h(n) \ge n$, this implies that $\varepsilon _n^2 n \rightarrow \infty$. Since $\psi (n, \varepsilon _n) \ge 0$, we have that $(\varepsilon _n + \psi (n, \varepsilon _n) ) / n^{-1/2} \rightarrow \infty$, i.e., the game is not 1/2-transient. In conclusion, if a game is $\delta$-transient, then $\delta < 1/2$.

##### Remark 5

A sufficient condition under which a directed game is $\delta$-transient is the following: there exist real numbers $\alpha \in [0, 2 - 2 \delta )$ and $\beta \in [0, 2 - 2 \delta - \alpha )$ such that $h(n) = O(n^{\alpha })$ and $\max _{z\in Z} |Z^{(n)}(z)| = \exp (O(n^{\beta }))$.

Note that the definition of a $\delta$-transient game is independent of the payoffs and only makes assumptions on the state space. In Sect. 2.1.1, we give a few examples of $\delta$-transient games.

**Oriented games.** Oriented games were introduced by Garnier and Ziliotto [12] and are defined as follows. Fix an integer $d \ge 1$, and denote by $e_i$ the *d*-dimensional vector with 1 in coordinate *i* and 0 in all other $d - 1$ coordinates. Given positive integers $n_1,\ldots ,n_d\ge 1$, a directed graph $\Gamma$ with vertex set $Z\subseteq \mathbb Z^d$ is called $(n_1,\ldots ,n_d)$-*invariant* (or simply *invariant*) if, for every $i \in [1, d]$, the translation at vector $n_i e_i$ is a graph isomorphism for $\Gamma$. A directed weakly transitive game is called *oriented* if its underlying graph $\Gamma$ is invariant and there exists $u \in \mathbb {R}^d\setminus \{0\}$ such that, for every directed edge (*z*, *w*) in $\Gamma$, we have $(w-z) \cdot u > 0$ (here, $\cdot$ denotes the usual scalar product of vectors in $\mathbb R^d$). We show the following proposition by providing a simple explicit construction.

##### Proposition 6

Every oriented game is $\delta$-transient for all $\delta \in (0,1/2)$.

The following two classes of games present particular examples of oriented games.

##### Example 7

**Fig. 1:**
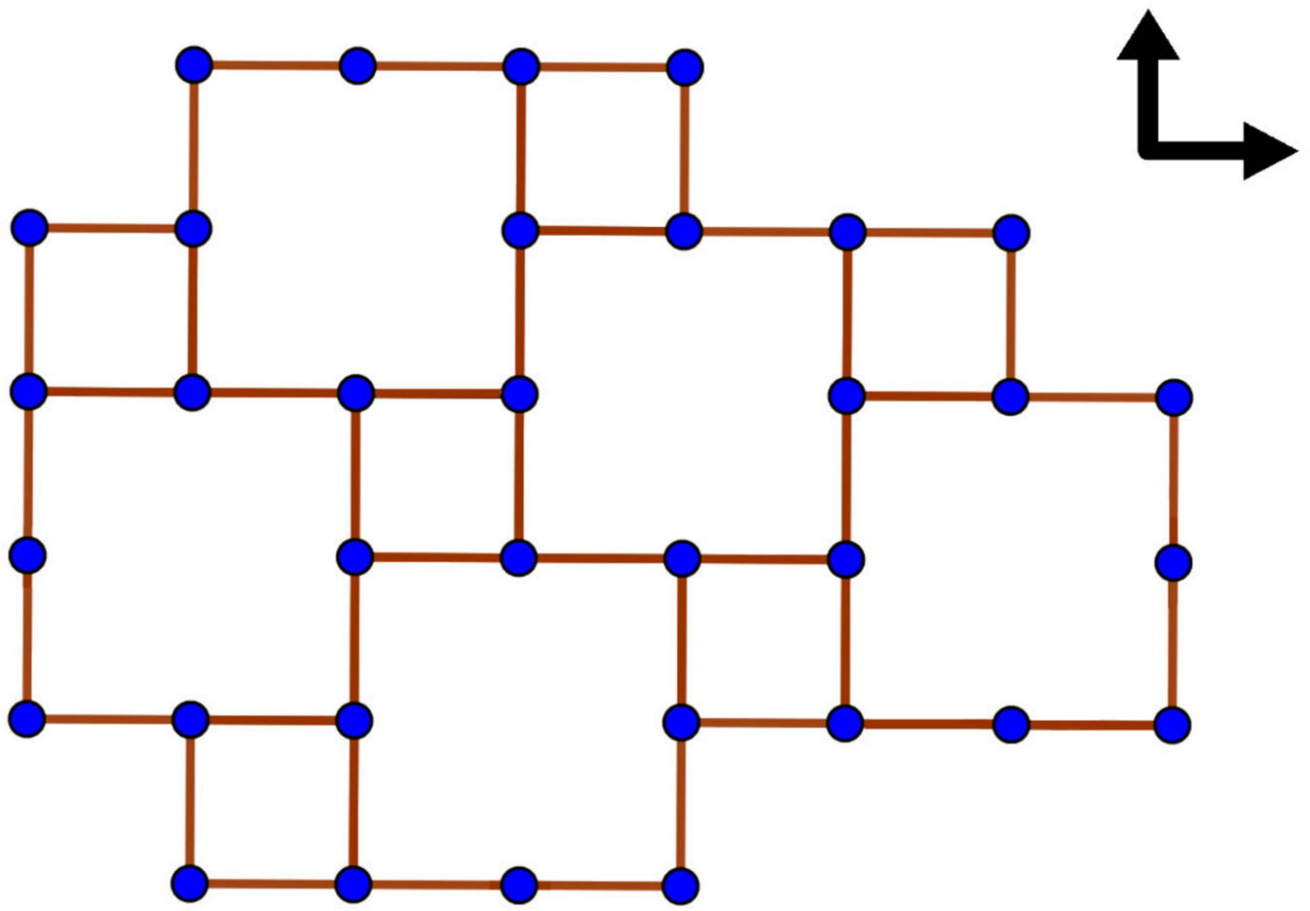
The figure depicts part of a tiling with two types of square tiles. The vertices and the edges of the planar graph originating from the tiling are depicted in blue and red, respectively. Each horizontal edge is oriented from left to right and every vertical edge is oriented from bottom to top. One may choose $z^*$ to be the bottom left vertex of a small square and $M=6$

(Games on tilings, see Fig. 1) A *tiling* is a periodic partition of the plane into translations of one or several polygonal shapes, called *tiles*, with vertices in $\mathbb Z^2$. Tilings naturally define planar graphs whose vertex set coincides with the corners of the tiles and two vertices are connected by an edge if these can be connected by following the boundary of a tile without meeting another vertex on the way. Equipping the edges of this graph with suitable orientations defines an oriented game.

##### Example 8

(Games on directed chains of graphs) Fix a finite vertex-transitive graph *H* with vertex set *V*(*H*) and edge set *E*(*H*), and a bi-infinite sequence of copies $(H_i)_{i \in \mathbb Z}$ of *H*. For every $i\in \mathbb Z$ and $u\in V(H)$, denote by $u_i$ the vertex in $H_i$ corresponding to *u*. We call an *H*-*chain* the graph $\Gamma _H$ with vertices $\bigcup _{i\in \mathbb Z} V(H_i)$ and edges $\{u_iv_{i+1}: i\in \mathbb Z, uv\in E(H)\}$.

Games on *H*-chains can be seen as instances of oriented games on $\mathbb Z$. Indeed, fixing $h = |V(H)|$, one may identify the vertices of $H_i$ with the integers in the interval $[ih+1, (i+1)h]$ for all $i\in \mathbb Z$ in a translation-invariant way.

**Weakly transitive games with controlled expansion.** In the following paragraphs, we show that one can construct a tree *T* with an arbitrary growth that is faster than linear but slower than exponential. In particular, we have the following result.

##### Proposition 9

For every $\delta \in (0, 1/2)$, there exist games that are $\delta$-transient but are not $\delta '-$transient for every $\delta ' > \delta$.

##### Proof

Fix an arbitrary infinite rooted tree *T* with root *r* and a family of vertex-disjoint infinite paths $(P_v)_{v \in V(T)}$ where the path $P_v$ intersects *T* at a unique vertex *v*. Define $\Gamma = T \cup (\bigcup _{v\in V(T)} P_v)$ as the tree rooted in *r* and with all edges oriented away from *r*. Thus, $\Gamma$ is a directed rooted tree larger than *T*. Since a single move of each player is sufficient to place the token at the second vertex of some infinite path among $(P_v)_{v \in V(T)}$, the game is weakly transitive.

Recall that $Z_n(z)$ consists of all vertices at distance *n* from *z*. Therefore, the sequence $(Z_n(r))_{n \ge 1}$ is a partition of the vertex set *Z* of $\Gamma$ and $Z_n(r)$ can only be visited once. Since $Z^{(n)}(r)$ can be partitioned into *n* antichains $Z_1, Z_2, \ldots , Z_n$, we have that $h(n, r) \le n$.

Let us show that we can control the growth speed of $\max _{z\in Z} |Z^{(2n)}(z)|$. Consider a set of non-negative integers $L = \{\ell _i: i\ge 1\}$ with $\ell _1< \ell _2 < \ldots$ and let every vertex of *T* in level $\ell$ have two children if $\ell \in L$ and one child otherwise. Moreover, suppose that $\ell _1 = 0$ and $(\ell _i - \ell _{i-1})_{i \ge 1}$ is a non-decreasing sequence. Then, one can readily check that, for every $n\ge 1$, $\max _{z\in Z} |Z^{(n)}(z)| = |Z^{(n)}(r)|$, and $h(n, z) = h(n, r) = n$. Indeed, for every $k, n\ge 1$ and a vertex $z\in Z$ on level *k*, using the assumptions that $\ell _1 = 0$ and $(\ell _i - \ell _{i-1})_{i \ge 1}$ is a non-decreasing sequence, we get

$$\begin{aligned}|Z^{(n)}(z)\setminus Z^{(n-1)}(z)| = 2^{|L\cap \{k,\ldots ,k+n-1\}|}\le 2^{|L\cap \{0,\ldots ,n-1\}|} = |Z^{(n)}(r)\setminus Z^{(n-1)}(r)|.\end{aligned}$$

Thus, for every integer $n\ge 0$, $|Z^{(n)}(r)| = 1+\sum _{i=0}^{n-1} 2^{|L\cap \{0,\ldots ,i\}|}$. $\square$

#### Directed Games on *d*-ary Trees

We turn our attention to a natural example of a directed game where the set of reachable states after *n* steps grows exponentially with *n*. Note that, for all $\delta > 0$, it is not a $\delta$-transient game. Fix an integer $d\ge 2$ and let *T* be an *infinite d-ary tree*, that is, a tree where every vertex has *d* children, with vertex set *Z* where every edge is directed from the parent to the child. We fix an arbitrary initial vertex $z_0$ and, for every integer $i \ge 0$, we define $Z_i$ to be the set of vertices in *Z* that can be reached from $z_0$ by exactly *i* steps and also denote $Z_\mathrm{{even}}:= \bigcup _{i\ge 0} Z_{2i}$ and $Z_\mathrm{{odd}}:= \bigcup _{i\ge 0} Z_{2i+1}$. Note that, for every $n \ge 1$, the random variables $(V_n(z))_{z \in Z}$ have the same distribution. Hence, in this setting, we often omit the dependence of $V_n$ in *z*.

### Main Results

Our first main result shows sharp concentration for the *n*-stage value of $\delta$-transient games around a deterministic constant.

#### Theorem 10

Fix $\delta \in (0,1/2)$. Consider a $\delta$-transient directed game with transient speed *h* and i.i.d. payoffs $(G_z)_{z \in Z}$ supported on the interval [0, 1]. Then, there exist constants $v_\infty \in [0, 1]$ and $K > 0$ such that, for all $n\ge 1$, $t\ge 0$, and $z \in Z$,

$$\begin{aligned} \mathbb {P}\left( |V_n(z) - v_\infty | \ge t + K n^{-\delta } \right) \le 2 \exp \left( -\frac{t^2 n^2}{2h(n)} \right) . \end{aligned}$$

Consequently, for all $z \in Z$, $(V_n(z))_{n \ge 1}$ converges almost surely to $v_\infty$.

Our second main result shows that the *n*-stage value of the directed game on a *d*-ary tree is tightly concentrated around a constant.

#### Theorem 11

Fix an integer $d\ge 2$. Consider a directed game on the *d*-ary tree with i.i.d. payoffs supported on the interval [0, 1]. Then, there exists a constant $v_\infty \in [0, 1]$ such that, for every $\delta \in (0,1/2)$, there exists $K > 0$ such that, for all $n \ge 1$, $t \ge 0$, and $z \in Z$,

$$\begin{aligned} \mathbb {P}(|V_n(z) - v_\infty | \ge t + 2t^2 + Kn^{-\delta }) \le \exp \left( -\frac{1}{6}\exp \left( \frac{t^2n}{4}\right) \right) . \end{aligned}$$

Consequently, for all $z \in Z$, $(V_n(z))_{n \ge 1}$ converges almost surely to $v_\infty$.

On a high level, the proofs of both theorems contain two main steps.

1. The first step relies on concentration arguments showing that $V_n$ is close to $\mathbb E[V_n]$ with high probability. While standard concentration tools are sufficient for our proof of Theorem 10, the stronger probabilistic bound in Theorem 11 requires an additional boosting obtained by dividing the first *n* levels of the *d*-ary tree into two groups of consecutive levels and treating the *n*-stage game as two consecutive games on *k* and $n-k$ stages, respectively.
2. The second step uses the structure of the underlying graph to show that $(\mathbb E[V_n])_{n \ge 1}$ satisfies a certain subadditivity assumption, which allows us to conclude that $(\mathbb E[V_n])_{n \ge 1}$ converges to a constant $v_{\infty }$, and moreover, $|\mathbb E[V_n]-v_{\infty }|$ is polynomially small.

***Perspectives.*** The proofs of Theorems 10 and 11 have a similar structure but use different arguments. A challenging research question would be to prove convergence of $(V_n)_{n \ge 1}$ and concentration bounds in all weakly transitive directed games, irrespective of the expansion speed of the underlying graph, thus unifying Theorems 10 and 11.

## Preliminary Results

In our proofs, we make use of the well-known *bounded difference inequality*, also known as *McDiarmid’s inequality*.

### Lemma 12

([24, Corollary 2.27]) Fix a function $f :\Lambda _1 \times \dots \times \Lambda _N \rightarrow \mathbb {R}$ and let $Y_1, \dots , Y_N$ be independent random variables taking values in $\Lambda _1, \dots , \Lambda _N$, respectively. Suppose that there are positive constants $c_1, \dots , c_N$ such that, for every two vectors $z, w \in \Lambda _1\times \dots \times \Lambda _N$ that differ only in the *k*-th coordinate, we have $|f(z) -f(w)| \le c_k$. Then, for every $t \ge 0$, the random variable $X = f(Y_1,\dots ,Y_N)$ satisfies

$$\begin{aligned} \mathbb {P}(X - \mathbb {E}[X] \ge t) \le \exp \left( -\frac{t^2}{2\sum _{i=1}^N c_i^2}\right) . \end{aligned}$$

$$\begin{aligned} \mathbb {P}(X - \mathbb {E}[X] \le -t) \le \exp \left( -\frac{t^2}{2\sum _{i=1}^N c_i^2}\right) . \end{aligned}$$

We also use the following result that states convergence of almost subadditive sequences.

### Lemma 13

([25, Theorem 23]) Fix an increasing function $\phi :\mathbb {N}\rightarrow (0, \infty )$ such that the sum of $(\phi (n)/n^2)_{n \ge 1}$ is finite, and a function $f :\mathbb {N}\rightarrow \mathbb {R}$ such that, for all $n \in \mathbb {N}$ and all integers $m \in [n/2, 2n]$, $f(n + m) \le f(n) + f(m) + \phi (n + m)$. Then, there exists $\ell \in \mathbb {R}\cup \{-\infty \}$ such that

$$\begin{aligned} \left( \frac{f(n)}{n} \right) \xrightarrow [n \rightarrow \infty ]  \ell . \end{aligned}$$

## $\varvec{\delta }$-transient Games: Proof of Theorem 10

To begin with, by using a suitable partition of the state space into subsets that are visited at most once, we show that the value of the *n*-stage game is well concentrated around its expected value. Note that the next lemma holds for weakly transitive games in general and will be reused in Sect. 5.

### Lemma 14

For every $z_0\in Z$, $n \ge 1$, and $t \ge 0$,

$$\begin{aligned} \mathbb {P}(V_n(z_0) - \mathbb {E}[V_n(z_0)]\ge t)\le \exp \left( -\frac{t^2n^2}{2 h(n)}\right) , \end{aligned}$$

$$\begin{aligned} \mathbb {P}(V_n(z_0) - \mathbb {E}[V_n(z_0)]\le -t)\le \exp \left( -\frac{t^2n^2}{2 h(n)}\right) . \end{aligned}$$

### Proof

Let us fix $z_0\in Z$ and abbreviate $V_n = V_n(z_0)$, $Z_n = Z_n(z_0)$ and $Z^{(n)} = Z^{(n)}(z_0)$. Define the (random) vectors $X_k = (G_z)_{z \in Z_k}\in [0,1]^{|Z_k(z_0)|}$. Then, since $Z^{(n)}\subseteq Z_{[h(n)]}$, $V_n$ can be written as $f(X_1, \dots , X_{h(n)})$ for some function $f :[0,1]^{|Z_1|}\times \dots \times [0,1]^{|Z_{h(n)}|}\rightarrow \mathbb {R}$. Moreover, for every integer $k\in [1, h(n)]$, the token visits the set $Z_k$ at most once and therefore, for every pair of strategies $(\sigma , \tau )\in \Sigma \times {\mathcal {T}}$, $\gamma _n^{z_0}(\sigma , \tau )$ varies by at most 1/*n* as a function of $X_k$. Hence, for every choice of vectors $(x_i)_{i = 1}^{h(n)} \in [0,1]^{|Z_1|} \times \dots \times [0,1]^{|Z_{h(n)}|}$ and $x_k'\in [0,1]^{|Z_k|}$,

$$\begin{aligned} |f(x_1, \dots , x_k, \dots , x_{h(n)}) - f(x_1, \dots , x_k', \dots , x_{h(n)})| \le \frac{1}{n}. \end{aligned}$$

Lemma 12 applied to $V_n$ finishes the proof. $\square$

In the remainder of the proof, we show that the expected *n*-stage value converges to a constant polynomially fast. Next, we show how to control the difference of the values of games of different lengths.

### Lemma 15

Fix integers $n\ge 1$ and $k\in [1,n]$. Then, for every $z_0\in Z$, $|V_n(z_0)-V_{n-k}(z_0)| \le k/n$.

### Proof

Observe that $nV_{n}(z_0) \ge (n-k)V_{n-k}(z_0)$ and $nV_{n}(z_0) \le (n-k)V_{n-k}(z_0) + k$. Indeed, in the *n*-stage game, Player 1 (respectively Player 2) may first play according to an optimal strategy for the $(n-k)$-stage game, and play arbitrarily during the last *k* stages. Hence, $|n(V_n(z_0) - V_{n-k}(z_0))|\le \max (kV_{n-k}(z_0), k-kV_{n-k}(z_0))\le k$, which implies the statement of the lemma. $\square$

The next lemma shows that starting from different initial states changes the expected *n*-stage value only slightly when *n* is large.

### Lemma 16

Consider a graph $\Gamma$ and denote by $z^*$ a state by which $\Gamma$ is weakly transitive. For every initial state $z_0\in Z$, we have that $|\mathbb E[V_n(z_0)] - \mathbb E[V_n(z^*)]| = O(n^{-\delta })$.

The main ideas of the proof are as follows: Given states $z,z^*$, the assumption of weak transitivity allows us to bound from below (respectively from above) $V_n(z)$ using the minimum (respectively the maximum) of the *n*-stage values over the nearby states equivalent to $z^*$. Moreover, by $\delta$-transience, the graph $\Gamma$ does not expand too quickly, which implies that, by a union bound, the expectation of the minimum (respectively the maximum) of the said *n*-stage values is approximately equal to the minimum (respectively the maximum) of their expectations.

### Proof of Lemma 16

Fix an initial state $z_0\in Z$ and denote by *E* the set of states in $Z^{(M)}(z_0)$ that are equivalent to $z^*$. By Definition 2, $E \ne \emptyset$ and, independently of the moves of Player 2, Player 1 can ensure that the token is at a state in *E* after an even number of $\ell \le M$ stages. Hence, using Lemma 15, we have

1 $$\begin{aligned} nV_n(z_0) \ge (n-M)\min _{z\in E} V_{n-M}(z) \ge (n-M)\min _{z\in E}(V_n(z)-M/n) \ge \min _{z\in E} nV_n(z) - 2M . \end{aligned}$$

Now, we bound from below the expectation of the right-hand side. Combining Lemma 14 and the choice of $\varepsilon _n$ from Definition 3, we have

2 $$\begin{aligned} \begin{aligned} \mathbb E\left[ \min _{z\in E} V_n(z)\right]&\ge (\mathbb E[V_n(z^*)] - \varepsilon _n) (1-\mathbb P(\exists z\in E: V_n(z)\le \mathbb E[V_n(z)]-\varepsilon _n))\\&\ge (\mathbb E[V_n(z^*)] - \varepsilon _n) \bigg (1-\exp \bigg (-\frac{\varepsilon _n^2n^2}{2h(n)}\bigg ) \max _{z\in Z} |Z^{(M)}(z)|\bigg )\\&\ge (\mathbb E[V_n(z^*)] - \varepsilon _n) (1-\psi (n, \varepsilon _n)) = \mathbb E[V_n(z^*)] - O(n^{-\delta }) , \end{aligned} \end{aligned}$$

where the second inequality comes from a union bound, the third one uses that $Z^{(M)}(z)\subseteq Z^{(2n)}(z)$ for every $z\in Z$, and the equality is implied by the fact that $\varepsilon _n+\psi (n, \varepsilon _n) = O(n^{-\delta })$. Thus, taking expectations on both sides of (1) and using (2) shows that

3 $$\begin{aligned} \mathbb E[V_n(z_0)]\ge \mathbb E[V_n(z^*)] - O(n^{-\delta }+2M/n) = \mathbb E[V_n(z^*)] - O(n^{-\delta }) . \end{aligned}$$

Similarly, Player 2 can ensure that the token reaches a state in *E* after an even number of $\ell \le M$ stages. Hence,

4 $$\begin{aligned} nV_n(z_0)\le (n-M)\max _{z\in E} V_{n-M}(z)+M \le \max _{z\in E} nV_n(z)+M . \end{aligned}$$

At the same time, similarly to (2), $\mathbb E\left[ \max _{z\in E} V_n(z)\right]$ is bounded from above by

$$\begin{aligned} (\mathbb E[V_n(z^*)] + \varepsilon _n) (1-\mathbb P(\exists z\in E: V_n(z) \ge \mathbb E[V_n(z)]+\varepsilon _n)) \\ + \mathbb P(\exists z\in E: V_n(z)\ge \mathbb E[V_n(z)]+\varepsilon _n) , \end{aligned}$$

which is at most $\mathbb E[V_n(z^*)] + (\varepsilon _n+\psi (n, \varepsilon _n)) = \mathbb E[V_n(z^*)] + O(n^{-\delta })$. Combining this with (4) shows that $\mathbb E[V_n(z_0)]\le \mathbb E[V_n(z^*)]+O(n^{-\delta })$, and, together with the lower bound in (3), this finishes the proof. $\square$

Next, we show that the expected value of $V_n$ converges as $n\rightarrow \infty$.

### Lemma 17

There is a constant $v_{\infty }$ such that, $|\mathbb {E}[V_n(z)] - v_{\infty }| = O(n^{-\delta })$, for every $z \in Z$.

As before, we start with an outline of the proof. We first use Lemma 16 to show that the initial state of the game is not important. Then, we justify that the sequence $(-n \mathbb {E}[V_n(z)])_{n \ge 1}$ is approximately subadditive, and use Lemma 13. The subadditivity is based on the simple observation that, for every player, playing optimally in the $(m+n)$-stage game is better than playing optimally in the initial *m*-stage game (based only on the *m*-th neighborhood of the initial state), and then do the same in the subsequent *n*-stage game.

### Proof of Lemma 17

By Lemma 16, it is sufficient to show the lemma when $z_0 = z^*$; we abbreviate $V_n = V_n(z^*)$ and $Z^{(n)} = Z^{(n)}(z^*)$ for convenience. First, we show that $\mathbb {E}[V_n]$ converges to a limit $v_{\infty }\in \mathbb R$ as $n\rightarrow \infty$. By Lemma 14 and a union bound, for all $t \ge 0$,

5 $$\begin{aligned} \begin{aligned} \mathbb {P}(\exists z \in Z^{(2n)}: |V_n(z) - \mathbb {E}[V_n(z)]| \ge t)&\le \sum _{z \in Z^{(2n)}} \mathbb {P}(|V_n(z) - \mathbb {E}[V_n(z)]| \ge t)\\&\le 2 \exp \left( -\frac{t^2 n^2}{2h(n)} \right) \max _{z\in Z} |Z^{(2n)}(z)| = 2\psi (n, t) , \end{aligned} \end{aligned}$$

where the factor of 2 in the last inequality appears since we bound both the upper and the lower tail of $V_n(z)$.

By definition of $\delta$-transient game, there exists $(\varepsilon _n)_{n \ge 1}$ such that $\varepsilon _n + \psi (n, \varepsilon _n) = O(n^{-\delta })$. Denote by *E* the set of vertices in $Z^{(2n)}$ that are equivalent to $z^*$. Now, Lemma 16 implies that there is a constant $K'>0$ such that, for every $z\in Z$ and $n\ge 1$, $|\mathbb E[V_n(z)]-\mathbb E[V_n]|\le K'n^{-\delta }$. Combining this with (5), we get that

$$\begin{aligned} \mathbb {P}\left( \min _{z \in Z^{(2n)}} V_n(z) \le \mathbb {E}[V_n] - \varepsilon _n - K'n^{-\delta } \right)&\le \mathbb {P}\left( \min _{z \in Z^{(2n)}} |V_n(z) - \mathbb {E}[V_n(z)]|\ge \varepsilon _n \right) \\&\le \mathbb {P}(\exists z \in E, |V_n(z) - \mathbb {E}[V_n(z)]| \ge \varepsilon _n) \\&\le 2\psi (n, \varepsilon _n) = O(n^{-\delta }) \,. \end{aligned}$$

In particular, it follows directly that

$$\begin{aligned} \mathbb {E}\left[ \min _{z \in Z^{(2n)}} V_n(z) \right]&\ge \left( \mathbb {E}[V_n] - \varepsilon _n - K'n^{-\delta } \right) \, \mathbb {P}\left( \min _{z \in Z^{(2n)}} V_n(z) \ge \mathbb {E}[V_n] - \varepsilon _n - K'n^{-\delta } \right) \\&\ge \left( \mathbb {E}[V_n] - \varepsilon _n - K'n^{-\delta } \right) \, (1-2\psi (n, \varepsilon _n)) \\&\ge \mathbb {E}[V_n] - 2(\psi (n, \varepsilon _n) + \varepsilon _n) - K'n^{-\delta } \,. \end{aligned}$$

Now, fix an integer $m \in [1,2n]$ and consider the $(m+n)$-stage game. Suppose that Player 1 plays according to an optimal strategy for the *m*-stage game up to stage *m* and, once the *m*-stage game terminates at a state $z_m$, continues to play according to an optimal strategy for the subsequent *n*-stage game. Note that $z_m\in Z^{(2n)}$, so the above strategy of Player 1 for the first $m+n$ steps guarantees a gain of $\tfrac{m}{m+n}V_m + \tfrac{n}{m+n}\min _{z \in Z^{(2n)}} V_n(z)$. Thus,

6 $$\begin{aligned} \begin{aligned} (m + n) \mathbb {E}[V_{m + n}]&\ge m \mathbb {E}[V_m] + n \mathbb {E}\left[ \min _{z \in Z^{(2n)}} V_n(z)\right] \\&\ge m \mathbb {E}[V_m] + n \mathbb {E}[V_n] - 2n(\psi (n, \varepsilon _n) + \varepsilon _n) - K'n^{1-\delta } . \end{aligned} \end{aligned}$$

Since $\psi (n, \varepsilon _n) + \varepsilon _n = O(n^{-\delta })$, there is a constant $K'' > 0$ such that, for all $n \ge 1$,

$$\begin{aligned} 2n(\psi (n, \varepsilon _n) + \varepsilon _n)+K'n^{1-\delta } \le 2K''n^{1-\delta } . \end{aligned}$$

Thus, using Lemma 13 with $f :n \mapsto -n \mathbb {E}[V_n]$ and $\phi :n\mapsto 2K'' n^{1 - \delta }$ (note that $\phi$ is increasing and $\sum _{n\ge 1} \phi (n) / n^2 = 2K'' \sum _{n\ge 1} 1 / n^{1 + \delta } < \infty$) implies that $\mathbb {E}[V_n]$ converges to a limit $v_{\infty }\in \mathbb R\cup \{\infty \}$ as $n\rightarrow \infty$. Note that $v_{\infty }$ is in [0, 1] since this is the support of all payoff variables.

Finally, using (6) with $m = n$, for every $n\ge 1$, we have that

$$\begin{aligned} \mathbb {E}[V_{2n}] \ge \mathbb {E}[V_n] - (\psi (n, \varepsilon _n) + \varepsilon _n) - \frac{K'n^{-\delta }}{2} \ge \mathbb {E}[V_n] - K'' n^{-\delta } . \end{aligned}$$

In particular, for all integers $\ell , n \ge 1$, iterating the above observation for *n* taking values $n, 2n, \dots , 2^{\ell -1}n$ gives that

7 $$\begin{aligned} \mathbb {E}[V_{2^\ell n}] \ge \mathbb {E}[V_n] - K'' n^{-\delta } \sum _{j=0}^{\ell -1} 2^{-\delta j} \ge \mathbb {E}[V_n] - \frac{K''}{1-2^{-\delta }}n^{-\delta } . \end{aligned}$$

Taking $\ell \rightarrow \infty$, we conclude that $v_\infty \ge \mathbb {E}[V_n] - O(n^{-\delta })$. A similar reasoning exchanging Player 1 with Player 2 shows that $v_\infty \le \mathbb {E}[V_n] + O(n^{-\delta })$ and concludes the proof of the lemma. $\square$

Finally, we are ready to prove Theorem 10.

### Proof of Theorem 10

Fix an arbitrary $\varepsilon > 0$, $z\in Z$ and let $V_n = V_n(z)$. By Lemma 17, there is a constant $K > 0$ such that $|v_\infty - \mathbb {E}[V_n]|\le K n^{-\delta }$ for all $n\ge 1$. Combining this with the triangle inequality and Lemma 14 shows that, for every $t\ge 0$,

$$\begin{aligned} \mathbb {P}( |V_n - v_\infty | \ge t + K n^{-\delta } )&\le \mathbb {P}( |V_n - \mathbb {E}[V_n]| \ge t + K n^{-\delta } - |\mathbb {E}[V_n] - v_\infty |) \\&\le \mathbb {P}( |V_n - \mathbb {E}[V_n]| \ge t) \le 2\exp \left( \frac{-t^2 n^2}{2h(n)} \right) \,, \end{aligned}$$

which is the desired result. $\square$

## Directed Games on Trees: Proof of Theorem 11

The first lemma in this section bootstraps upon the conclusion of Lemma 14 (which still holds in this setting), thus deriving superexponential concentration for the value of the *n*-stage game. Below, $\log$ stands for the natural logarithm.

### Lemma 18

Fix $\delta \in (0,1/2)$, $n\ge 1$ and $t\ge n^{-\delta }$. For every even integer $k\in [2,n]$ such that

8 $$\begin{aligned} k \log d + 2 \log 2 \le t^2 (n-k), \end{aligned}$$

we have

$$\begin{aligned} \mathbb {P}(nV_n - (n-k)\mathbb {E}[V_{n - k}] \ge&(n-k) t + k) \le \exp \left( -\frac{d^{k/2}}{6}\right) \,, \\ \mathbb {P}(nV_n - (n-k)\mathbb {E}[V_{n - k}] \le&-(n-k)t-k) \le \exp \left( -\frac{d^{k/2}}{6}\right) \,. \end{aligned}$$

The proof goes roughly as follows. Given an intermediate stage $k = k(n)$ of the game such that $(n - k(n))$ grows to infinity, there typically exist only a few vertices such that the value of the $(n-k)$-stage game starting from them has a value that is very far from the expectation (we see these vertices as “bad”). Therefore, with foresight, both players can avoid such “bad” vertices, thus boosting the concentration of the *n*-stage value.

### Proof of Lemma 18

First of all, since *T* is a transitive graph, for every fixed $n\ge 1$, the variables $(V_n(z))_z$ have the same distribution. For every even integer $k \in [n]$, denote

$$\begin{aligned} S_k:= \{ z \in Z_k: V_{n-k}(z)-\mathbb {E}[V_{n-k}] \ge t \} . \end{aligned}$$

In other words, $S_k$ is the set of vertices *z* that could be reached from $z_0$ after *k* stages, for which the value of the $(n-k)$-stage game starting at *z* is greater than or equal to $\mathbb {E}[V_{n-k}] +t$.

Define the event ${\mathcal {E}}_k:= \{|S_k| \ge d^{k/2}\}$. We provide an upper bound for $\mathbb {P}({\mathcal {E}}_k)$. Since the random variables $(V_{n-k}(z))_{z \in Z_k}$ are i.i.d., we have that $|S_k|$ follows a binomial distribution $\textrm{Bin}(d^k, q)$ where $q:= \mathbb {P}(V_{n-k} \ge \mathbb {E}[V_{n-k}] + t)$. Consequently, by Lemma 14 (where the transient speed *h* is the identity function on $\mathbb {N}$ since, for all $z\in Z$ and $k\ge 2$, $Z_k(z)$ consists of all descendants of *z* at distance *k*, which form an antichain), $|S_k|$ is stochastically dominated by a binomial random variable $\textrm{Bin}(d^k, {\tilde{q}})$ where ${\tilde{q}} = \exp (-t^2(n-k)/2)$. In particular,

$$\begin{aligned} \mathbb {P}({\mathcal {E}}_k) \le \mathbb {P}\left( \textrm{Bin}(d^k, {\tilde{q}}) \ge d^{k/2} \right) . \end{aligned}$$

The random variable $\textrm{Bin}(d^k, {\tilde{q}})$ has mean $\mu := d^k {\tilde{q}}$. We define

$$\begin{aligned} \xi := \frac{d^{k/2}}{\mu } - 1 = \exp \left( \frac{t^2(n-k) - k\log (d)}{2}\right) - 1 \ge 1 , \end{aligned}$$

where the last inequality comes from (8). Since $d^{k/2} = (1 + \xi ) \mu$, we have that

$$\begin{aligned} \mathbb {P}( \textrm{Bin}(d^k, {\tilde{q}}) \ge d^{k/2} ) = \mathbb {P}( \textrm{Bin}(d^k, {\tilde{q}}) \ge (1+\xi )\mu ) . \end{aligned}$$

Therefore, since $\xi \ge 1$ (so $3\xi \ge 2+\xi$), by Chernoff’s bound,

$$\begin{aligned} \mathbb {P}\left( \textrm{Bin}(d^k, {\tilde{q}}) \ge d^{k/2} \right)&\le \exp \left( -\frac{\xi ^2\mu }{2+\xi }\right) \\&\le \exp \left( -\frac{\xi \mu }{3}\right) \\&= \exp \left( -\frac{d^{k/2}}{3} \left( 1-d^{k/2}{\tilde{q}}\right) \right) \,. \end{aligned}$$

Since $\xi = 1/(d^{k/2}{\tilde{q}}) - 1\ge 1$, we have that $1-d^{k/2}{\tilde{q}} \ge 1/2$, which finally yields

9 $$\begin{aligned} \mathbb {P}({\mathcal {E}}_k) \le \exp \left( -\frac{d^{k/2}}{6} \right) . \end{aligned}$$

At the same time, on the event $|S_k| < d^{k/2}$ (that is, $\overline{{\mathcal {E}}_k}$), Player 2 can ensure that the token avoids ending up in $S_k$ after *k* stages. Indeed, at each of the $k/2\in \mathbb N$ turns corresponding to decisions of Player 2, by the pigeonhole principle, Player 2 can always move the token to a vertex having at most a (1/*d*)-fraction of all remaining elements in $S_k$ among its descendants. Since Player 2 has *k*/2 turns and $d^{-k/2} |S_k| < 1$, Player 2 can safely avoid the set $S_k$ at stage *k*.

Let us condition on the event $\overline{{\mathcal {E}}_k}$. Then, Player 2 can guarantee that the sum of the payoffs over the last $n-k$ stages is strictly smaller than $(n-k)(\mathbb {E}[V_{n-k}] + t)$. Moreover, the sum of the first *k* payoffs is at most *k*. Consequently, Player 2 can guarantee that, after *n* stages, the global mean payoff is strictly smaller than $k/n + (n-k)(\mathbb {E}[V_{n-k}] + t)/n$, in other words,

10 $$\begin{aligned} nV_n < (n-k) \mathbb {E}[V_{n - k}] + (n-k) t + k . \end{aligned}$$

In particular, using (9) implies that

$$\begin{aligned} \mathbb {P}\left( nV_n - (n-k) \mathbb {E}[V_{n - k}] \ge (n-k) t + k \right) \le \mathbb {P}(|S_k| \ge d^{k/2}) = \mathbb {P}({\mathcal {E}}_k) \le \exp \left( -\frac{d^{k/2}}{6} \right) . \end{aligned}$$

A similar reasoning for Player 1 (using the sets ${\tilde{S}}_k:= \{ z \in Z_k: V_{n-k}(z)-\mathbb {E}[V_{n-k}] \le -t \}$ instead of $S_k$ and replacing (10) with $nV_n > (n-k)\mathbb E[V_{n-k}] - (n-k)t$) yields

$$\begin{aligned} \mathbb {P}\left( nV_n - (n-k)\mathbb {E}[V_{n - k}] \le -(n-k)t\right) \le \exp \left( -\frac{d^{k/2}}{6} \right) , \end{aligned}$$

which implies the second statement. Note that the additional $-k$ in it is introduced for reasons of symmetry only. $\square$

Next, we show that the expected value of the *n*-stage game converges rapidly as *n* grows to infinity.

### Lemma 19

There exists $v_\infty \in \mathbb {R}$ such that, for every $\delta \in (0,1/2)$, we have $|\mathbb {E}[V_n] - v_\infty | = O(n^{-\delta })$.

The arguments in the proof are very similar to the ones from the proof of Theorem 17 but the stronger concentration derived in Lemma 18 replaces the standard bounded difference estimate from Lemma 14.

### Proof

Fix $\delta ' \in (0,1/2)$, $n\ge 1$, and $t\ge n^{-\delta '}$. Set $k = k(n):= 2 \big \lfloor n^{1-2\delta '}/4\log d \big \rfloor$. Then, $k\log d + 2 \log 2 \le t^2 (n-k)$ for all large *n*. For every even integer $m \in [n/2,2n]$ and large *n*, we have

$$\begin{aligned}&\mathbb {P}\left( \min _{z \in Z_{m}} nV_n(z) \le (n-k)(\mathbb {E}[V_{n-k}]-t) - k \right) \\&\qquad \le \sum _{z \in Z_{m}} \mathbb {P}\left( nV_n(z) \le (n-k)(\mathbb {E}[V_{n-k}]-t) - k \right) \\&\qquad \le d^m \exp \left( -\frac{d^{k/2}}{6} \right) \\&\qquad \le \exp \left( 2n\log d -\frac{d^{\lfloor n^{1-2\delta '}/4\log d\rfloor }}{6} \right) \,, \end{aligned}$$

where the first inequality comes from a union bound and the second inequality comes from Theorem 18. Fix $\delta \in (0, \delta ')$ and define, for all $n \ge 1$,

$$\begin{aligned} \varepsilon _n:= n^{-\delta } \quad \text {and}\quad \psi (n):= \exp \left( 2n\log d -d^{\lfloor n^{1-2\delta '}/4\log d\rfloor }/6 \right) . \end{aligned}$$

For large *n* and every even integer $m \in [n/2, 2n]$, we have

11 $$\begin{aligned} \mathbb {E}\left[ \min _{z \in Z_{m}} V_n(z) \right]&\ge \left( \frac{n-k}{n}(\mathbb {E}[V_{n-k}]-\varepsilon _n) - \frac{k}{n}\right) \end{aligned}$$

12 $$\begin{aligned}&\qquad \mathbb {P}\left( \min _{z \in Z_{m}} nV_n(z) > (n-k)(\mathbb {E}[V_{n-k}]-\varepsilon _n) - k \right) \nonumber \\&\ge \left( \frac{n-k}{n}(\mathbb {E}[V_{n-k}]-\varepsilon _n) - \frac{k}{n}\right) (1- \psi (n))\nonumber \\&\ge \left( \mathbb {E}[V_{n-k}] - \frac{k}{n}(1+\mathbb {E}[V_{n-k}]) - \varepsilon _n \right) (1 - \psi (n))\nonumber \\&\ge \left( \mathbb {E}[V_{n}] - \frac{3k}{n} - \varepsilon _n \right) (1 - \psi (n)) \ge \mathbb {E}[V_n] - (\psi (n) + 2\varepsilon _n) \,, \end{aligned}$$

where in the fourth inequality we used that $\mathbb E[V_n]\le \mathbb E[V_{n-k}]+k/n$ by Lemma 15 and $1+\mathbb E[V_{n-k}]\le 2$, and the last inequality is valid for large *n* because $k/n = o(\varepsilon _n)$.

Consider integers $n \ge 1$ and even $m\in [n/2,2n]$. In the $(n+m)$-stage game, Player 1 can play according to an optimal strategy for the *m*-stage game starting at $z_0$, and then play according to an optimal strategy for the *n*-stage game starting from the state *z* reached after *m* stages. This guarantees that $(m+n)V_{m+n}\ge m V_m + \min _{z \in Z_m} n V_n(z)$. Taking expectations on both sides and using (12) yields

$$\begin{aligned} (m + n) \mathbb {E}[V_{m + n}]&\ge m \mathbb {E}[V_m] + n \mathbb {E}\left[ \min _{z \in Z_m} V_n(z) \right] \\&\ge m \mathbb {E}[V_m]+ n\mathbb {E}[V_n] - n(\psi (n) + 2\varepsilon _n) \,. \end{aligned}$$

We find a similar inequality for odd $m\in [n/2,2n]$. In this case, $m+1$ is even and also in [*n*/2, 2*n*]. Then, the previous inequality applied to $m+1$ and *n* yields

13 $$\begin{aligned} (m+n+1) \mathbb {E}[V_{m+n+1}] \ge (m+1) \mathbb {E}[V_{m+1}]+ n\mathbb {E}[V_n] - n(\psi (n) + 2\varepsilon _n) . \end{aligned}$$

However,

$$\begin{aligned} (m+n) \mathbb {E}[V_{m+n}] \ge (m+n+ 1) \mathbb {E}[V_{m+n+1}]-1 \quad \text {and}\quad (m+1) \mathbb {E}[V_{m+1}] \ge m \mathbb {E}[V_{m}] , \end{aligned}$$

which combined with (13) gives

$$\begin{aligned} (m + n) \mathbb {E}[V_{m+n}] \ge m \mathbb {E}[V_{m}]+ n\mathbb {E}[V_n] - n(\psi (n) + 2\varepsilon _n)-1 . \end{aligned}$$

To sum things up, for large *n* and $m\in [n/2,2n]$,

14 $$\begin{aligned} (m + n) \mathbb {E}[V_{m +n}] \ge m \mathbb {E}[V_{m}]+ n\mathbb {E}[V_n] - n(\psi (n) + 2\varepsilon _n) - 1 . \end{aligned}$$

Recall that there is a constant $K' > 0$ such that, for all $n\ge 1$, $n(\psi (n) + 2\varepsilon _n)+1 \le K' n^{1-\delta }$. We define $\phi (n):= K' n^{1-\delta }$ and deduce from (14) that

$$\begin{aligned} (m + n) \mathbb {E}[V_{m +n}] \ge m \mathbb {E}[V_{m}]+ n\mathbb {E}[V_n] - \phi (n+m) . \end{aligned}$$

Moreover, $\phi$ is increasing and verifies $\sum _{n\ge 1} \phi (n)/n^2 < \infty$. Consequently, Lemma 13 applied to the function $f :n\in \mathbb N\mapsto -n\mathbb E[V_n]$ implies that that $\mathbb {E}[V_n]$ converges to a limit $v_{\infty }\in \mathbb R\cup \{\infty \}$ as $n\rightarrow \infty$. Note that $v_{\infty }\in [0,1]$ since $V_n\in [0,1]$ for all $n\ge 1$.

Finally, using (14) with $m = n$ and a telescopic summation shows that the inequality (7) still holds. In particular, we conclude that $v_\infty \ge \mathbb {E}[V_n] - O(n^{-\delta })$. A similar reasoning replacing Player 1 with Player 2 shows that $v_\infty \le \mathbb {E}[V_n] + O(n^{-\delta })$ and concludes the proof of the lemma. $\square$

We are now ready to prove Theorem 11.

### Proof of Theorem 11

Fix $t\ge n^{-\delta }$ and let $K'$ be a constant such that $|\mathbb E[V_n] - v_{\infty }|\le K' n^{-\delta }$ for all large *n*. Using that, for all *n* and $k\le n$, we have $|nV_n - (n-k)V_{n-k}|\le k$, and fixing $k = 2\lceil \tfrac{t^2n}{4\log d}\rceil$ (which satisfies (8)), we get

$$\begin{aligned}&\mathbb {P}(|V_n - v_\infty |\ge t+2t^2+K' n^{-\delta }) \\&\qquad \le \mathbb {P}\bigg (\left| V_n - \frac{n-k}{n}\mathbb {E}[V_{n-k}]\right| \ge t+2t^2+K'n^{-\delta } \\&\qquad \qquad - \left| \frac{n-k}{n}\mathbb {E}[V_{n-k}] - \mathbb {E}[V_n]\right| - |\mathbb {E}[V_n]-v_\infty | \bigg ) \\&\qquad \le \mathbb {P}\left( \left| V_n - \frac{n-k}{n}\mathbb {E}[V_{n-k}]\right| \ge t + t^2 \right) \\&\qquad \le \mathbb {P}(|nV_n - (n-k)\mathbb {E}[V_{n - k}]| \ge (n-k)t + k) \\&\qquad \le \exp \left( -\frac{d^{\lfloor k/2\rfloor }}{6} \right) \le \exp \left( -\frac{d^{t^2n/(4\log d)}}{6} \right) = \exp \left( -\frac{1}{6}\exp \left( \frac{t^2n}{4}\right) \right) \,, \end{aligned}$$

where the first inequality comes from the triangle inequality, the second inequality comes from the definition of $K'$ and the fact that $|nV_n-(n-k)V_{n-k}|\le k\le nt^2$, and the third inequality once again uses the fact that $k\le nt^2$.

Finally, choosing $K\ge K'$ sufficiently large ensures that, first, the upper bound shown above holds for all $n\ge 1$ (and not only for large *n*), and second, the upper bound holds for all $t\ge 0$, which finishes the proof. $\square$

## Oriented Games: Proof of Proposition 6

We present a simple and self-contained Proof of Proposition 6.

### Proof of Proposition 6

First, by density of the rational vectors in $\mathbb {R}^d$ and rescaling, we may assume that $u \in \mathbb Z^d$ is such that the greatest common divisor of its coordinates is 1. We provide an upper bound on *h*(*n*). For every integer $i \ge 1$ and initial state $z_0 = z$, define $Z_{2i}(z):= \{ w \in Z: w \cdot u = z\cdot u + i \}$, $Z_{2 i + 1}(z):= \{ w \in Z: w \cdot u = z \cdot u - i \}$, and $Z_0(z):= \{ z \}$ and $Z_1(z):= \{ w \in Z \setminus \{ z \}: w \cdot u = z \cdot u \}$. Then, for all $z \in Z$, $(Z_i(z))_{i \ge 1}$ form a partition of *Z* and each of them can be visited at most once, i.e., for all $n \ge 1$, the sets $(Z_1, Z_2, \ldots , Z_n)$ forms an antichain. Set $r:= \max _{ (u, v) \in E(\Gamma ) } \Vert v - u \Vert _2$. After *n* steps of the game, the position $z_n$ satisfies $\Vert z_n - z \Vert _2 \le n r$, and by the Cauchy-Schwarz inequality,

$$\begin{aligned} |(z_n - z) \cdot u | \le \Vert z_n - z \Vert _2 \cdot \Vert u \Vert _2 \le \lceil n r \cdot \Vert u \Vert _2 \rceil =: N = N(n) . \end{aligned}$$

In particular, $Z^{(n)}(z)$ is contained in the ball with radius *N* around *z*, which itself is contained in $Z_1 \cup Z_2 \cup \ldots \cup Z_{2N + 1}(z)$, so the transient speed of the process satisfies $h(n) \le 2 N(n) + 1$ for all $n \ge 1$.

Now, fix $\delta \in (0, 1/2)$ and $z_0 = z \in Z$. We show that the game is $\delta$-transient. Set $\varepsilon _n:= n^{-\delta }$. Then,

$$\begin{aligned} \psi (n, \varepsilon _n)&= \exp \left( -\frac{\varepsilon _n^2 n^2}{2h(n)} \right) \max _{z\in Z}|Z^{(2n)}(z)|\\&\le \exp \left( -\frac{\varepsilon _n^2 n}{6 r\cdot \Vert u \Vert _2} \right) (2 n r \cdot \Vert u \Vert _2 + 1)^d \\&= \exp \left( -\frac{n^{1 - 2\delta }}{6 r\cdot \Vert u \Vert _2} \right) (2n r \cdot \Vert u \Vert _2 + 1)^d = O(n^{-\delta }) \,. \end{aligned}$$

Hence, for all $\delta \in (0, 1/2)$, $\varepsilon _n + \psi (n, \varepsilon _n) = O(n^{-\delta })$, and therefore, the game is $\delta$-transient. $\square$

## Data Availability

Not applicable.
